# Supplementary material for: High RRM2 expression has poor prognosis in specific types of breast cancer
Source: PLoS One. 2022 Mar 15;17(3):e0265195. doi: 10.1371/journal.pone.0265195 (PMC8923511; doi:10.1371/journal.pone.0265195)
Supplement: Attachment — Submitted filename: Response to Reviewers#2.docx [file pone.0265195.s009.docx]

Dear reviewer

Thank you for your letter and the reviewer's comments concerning our manuscript entitled " High RRM2 expression has poor overall survival in specific types of breast cancer"( Manuscript Number: PONE-D-21-23611). Your comments are valuable and very helpful. We have read through the comments carefully and have made corrections. We have uploaded the file of the revised manuscript. The responses to the reviewer's comments are presented following.

Sincerely.

Tao Wang

Reviewer #2: This manuscript demonstrates the role of RRM2 in breast cancer. Authors have shown that higher RRM2 of RRM2 is associated with a worse prognosis in breast cancer patients. They utilized publically available datasets such as KM plotters via utilizing univariate or multivariate analyses and GSEA analysis to show that RRM2 is indeed an important molecule for breast cancer progression and its higher expression is associated with ErbB and other cancer-associated signaling pathways. Overall, these studies suggest that RRM2 could be used as a prognostic factor for breast cancer patients. Although the findings are interesting and well organized, these studies are lacking some further details. RRM2 has been associated with drug resistance in other cancer types. Is RRM2 involved with chemoresistance in several breast cancer subtypes? It is also important to know if RRM2 is associated with distant metastasis-free survival. In addition, this reviewer would like to know if the gene expression data of RRM2 is also correlated with the proteomic data or protein atlas data.

Response:

To evaluate the effect of RRM2 expression level on distant metastasis-free survival (DMFS) in breast cancer patients, we performed Kaplan-Meier online survival analysis, and the results showed that, among all breast cancer patients, the RRM2 high expression group had worse DMFS (P < 0.05). We further evaluated the effect of RRM2 expression on DMFS in patients with different subtypes of breast cancer, and the results showed that in Luminal A breast cancer patients, high expression of RRM2 had worse DMFS (P < 0.05). However, The expression level of RRM2 has no significant correlation with the Basal-like subtype, the Luminal B subtype, basal-like subtype, and Her-2 enriched subtype. As shown in Fig 8.(line373-389)

We have confirmed that the relative mRNA expression level of RRM2 is up-regulated in breast cancer tissues and breast cancer cell lines by qRT-PCR and bioinformatics analysis. Next, we also hope to clarify whether there is a certain correlation between the RRM2 gene expression level and the protein expression level. We searched the [Human Protein Atlas](https://www.proteinatlas.org/) (HPA database) https://www.proteinatlas.org/, unfortunately, there are limited immunohistochemical data on RRM2 in breast cancer tissues. Then we detected the protein expression level of RRM2 by Western blot, and the results showed that the protein expression of RRM2 was significantly up-regulated in breast cancer tissues compared with adjacent normal tissues. At the same time, we also detected the protein expression level of RRM2 in breast cancer cell lines, and the same trend is obtained (Fig 2B-2C). Of course, more about RRM2 proteomics and protein changes in vital pathways in breast cancer need to be further explored.(line246-266)

RRM2 has been associated with drug resistance in other cancer types. Is RRM2 involved with chemoresistance in several breast cancer subtypes? In response to the above questions, we conducted a literature search. At present, the research of scholars mainly focuses on tamoxifen resistance (an endocrine therapy drug for breast cancer), and there is almost no research on chemoresistance in specific breast cancer subtypes. . We are very grateful for the opinions of the reviewers, which also provide a good direction and research ideas for our follow-up research. We need to design a feasible research protocol to further explore whether RRM2 affects chemoresistance in specific breast cancer subtypes.
